# Supplementary material for: Investigation of the canine elbow joint innervation in 100 joints
Source: PLoS One. 2025 Jan 27;20(1):e0316379. doi: 10.1371/journal.pone.0316379 (PMC11771925; doi:10.1371/journal.pone.0316379)
Supplement: S1 Table — (PDF) [file pone.0316379.s001.pdf]

```
+-----------------+
| Key             |
|-----------------|
|    frequency    |
| row percentage  |
+-----------------+

           |             median nerve
      size |         1          2          3 |     Total
-----------+---------------------------------+----------
       lar |         6          5          2 |        13 
           |     46.15      38.46      15.38 |    100.00 
-----------+---------------------------------+----------
       med |         8         12          5 |        25 
           |     32.00      48.00      20.00 |    100.00 
-----------+---------------------------------+----------
       sma |         6          2          4 |        12 
           |     50.00      16.67      33.33 |    100.00 
-----------+---------------------------------+----------
     Total |        20         19         11 |        50 
           |     40.00      38.00      22.00 |    100.00 
```
