# Supplementary material for: Investigation of the canine elbow joint innervation in 100 joints
Source: PLoS One. 2025 Jan 27;20(1):e0316379. doi: 10.1371/journal.pone.0316379 (PMC11771925; doi:10.1371/journal.pone.0316379)
Supplement: S2 Table — (PDF) [file pone.0316379.s002.pdf]

```
+-----------------+
| Key             |
|-----------------|
|    frequency    |
| row percentage  |
+-----------------+
```

	median nerve			
old	1	2	3	Total
age<=11	10	13	6	29
	34.48	44.83	20.69	100.00
age>11	10	6	5	21
	47.62	28.57	23.81	100.00
Total	20	19	11	50
	40.00	38.00	22.00	100.00
