# Supplementary material for: Investigation of the canine elbow joint innervation in 100 joints
Source: PLoS One. 2025 Jan 27;20(1):e0316379. doi: 10.1371/journal.pone.0316379 (PMC11771925; doi:10.1371/journal.pone.0316379)
Supplement: S3 Table — (PDF) [file pone.0316379.s003.pdf]

Key
frequency
row percentage

size	musculocutaneous nerve 1	2	3	Total
lar	0	13	0	13
	0.00	100.00	0.00	100.00
med	7	17	1	25
	28.00	68.00	4.00	100.00
sma	3	9	0	12
	25.00	75.00	0.00	100.00
Total	10	39	1	50
	20.00	78.00	2.00	100.00
