# Supplementary material for: Investigation of the canine elbow joint innervation in 100 joints
Source: PLoS One. 2025 Jan 27;20(1):e0316379. doi: 10.1371/journal.pone.0316379 (PMC11771925; doi:10.1371/journal.pone.0316379)
Supplement: S4 Table — (PDF) [file pone.0316379.s004.pdf]

Key
frequency
row percentage

old	musculocutaneous nerve 1	2	3	Total
age<=11	7	21	1	29
	24.14	72.41	3.45	100.00
age>11	3	18	0	21
	14.29	85.71	0.00	100.00
Total	10	39	1	50
	20.00	78.00	2.00	100.00
