# Supplementary material for: Investigation of the canine elbow joint innervation in 100 joints
Source: PLoS One. 2025 Jan 27;20(1):e0316379. doi: 10.1371/journal.pone.0316379 (PMC11771925; doi:10.1371/journal.pone.0316379)
Supplement: S5 Table — (PDF) [file pone.0316379.s005.pdf]

| Key |
|---|
| frequency |
| row percentage |

| size | radial nerve 1 | 2 | 3 | Total |
|---|---|---|---|---|
| lar | 11 | 0 | 2 | 13 |
| | 84.62 | 0.00 | 15.38 | 100.00 |
| med | 14 | 6 | 5 | 25 |
| | 56.00 | 24.00 | 20.00 | 100.00 |
| sma | 8 | 4 | 0 | 12 |
| | 66.67 | 33.33 | 0.00 | 100.00 |
| Total | 33 | 10 | 7 | 50 |
| | 66.00 | 20.00 | 14.00 | 100.00 |
