# Supplementary material for: Investigation of the canine elbow joint innervation in 100 joints
Source: PLoS One. 2025 Jan 27;20(1):e0316379. doi: 10.1371/journal.pone.0316379 (PMC11771925; doi:10.1371/journal.pone.0316379)
Supplement: S6 Table — (PDF) [file pone.0316379.s006.pdf]

Key
frequency
row percentage

old	radial nerve 1	2	3	Total
age<=11	20	5	4	29
	68.97	17.24	13.79	100.00
age>11	13	5	3	21
	61.90	23.81	14.29	100.00
Total	33	10	7	50
	66.00	20.00	14.00	100.00
