# Supplementary material for: Investigation of the canine elbow joint innervation in 100 joints
Source: PLoS One. 2025 Jan 27;20(1):e0316379. doi: 10.1371/journal.pone.0316379 (PMC11771925; doi:10.1371/journal.pone.0316379)
Supplement: Attachment — Submitted filename: Response to Reviewers.docx [file pone.0316379.s007.docx]

We appreciate reviewer’s comments. Thank you for your time and effort.

2.

The authors received no specific funding for this work.

3.

All relevant data are within the manuscript and its Supporting Information files.

Reviewer #1:

- The introduction is quite modest in the description of the studied nerves. In my opinion the description of the nerves should be developed with an indication of their sensory and motor functions, which would shed a light on the significance of these nerves and the study itself.

Corrections have been made to indicate the functions of motor and sensory nerves.

- In line 86, 151, 278… “The distribution of dogs with a different number…” may be understood that the dogs which have a different number of branches per joint are distributed differently than dogs without different number of branches… The Authors should consider changing the sentence. For example… The distribution/number of dogs in relation to the number of branches per joint.

The sentences have been modified.

- It should be pointed if the Figures are of own authorship, otherwise the source should be added

Photo sources have been added.

- In Fig.3. there should be a legend or description that a red line demonstrates the nerve

- In Figs 6, 9 and 11. same as above… the nerve branches should be marked

A legend has been prepared for the diagrams provided.

- Line 104… the abbreviation CI is not explained as confidence interval

This part was corrected.

- The statistical results of chi2 test should be presented in the table 3

This part was corrected.

- In lines 168 or 203 etc. I wound consider changing phrases like “results reported in [2]” into “results reported in Yilmaz and Bahadir’s study”

These parts have been modified

- In table 6, in one of the table column, there is untranslated fragment of the text

We translated that fragment of the text.

- In line 288, “respectively” is not necessary

The term has been removed.

- The References are not prepared in accordance to the PLOS ONE Submission Guidelines

This part was corrected.

Reviewer #2

Please remove the period at the end of the title.

This part was corrected.

Please remove the abbreviation "lat" throughout the manuscript (e.g. extensor carpi radialis muscle (lat: m. extensor carpi radialis) next to the names of anatomical structures-it is unnecessary. Please, next to the name in English write the name in Latin, the reader will know that it is Latin because it is given in italics.

All manuscript was modified.

Table 6, column 3 - please translate Polish into English.

We translated that fragment of the text.

I think that a weakness of this experience is a very modest list of publications to which the authors referred in preparing this manuscript.

One of the main motives of our research was the significantly small amount of literature data on the innervation of the canine elbow joint. This also affects the bibliography of the work. After receiving the reviews, we added several literature items to make the work more interesting.
