# Supplementary material for: Investigation of the canine elbow joint innervation in 100 joints
Source: PLoS One. 2025 Jan 27;20(1):e0316379. doi: 10.1371/journal.pone.0316379 (PMC11771925; doi:10.1371/journal.pone.0316379)
Supplement: Attachment — Submitted filename: Response to Reviewers.docx [file pone.0316379.s008.docx]

We appreciate reviewer’s comments. Thank you for your time and effort.

**The introduction information should include the description of the branches of each nerve to the elbow joint.**

The course of the nerves to the elbow joint was described according to the guidelines.

The introduction has been modified and the indicated titles of literature and articles on regional anesthesia of the nerves innervating the elbow joint have been included.

**The redaction in English and Latin should improve.**

This article has been edited in accordance with the Nomina Anatomica Veterinaria.

Suggestions for Latin names have been included in the article.

**I recommend changing the color of the nerve from red to yellow since this later is the most used in the anatomy veterinary textbooks (Figures 3, 6, 9, 11)**

The colors of the marked nerves have been changed as suggested.

**The photographs of the figures are blurred (Figures 2, 5, 7, 8, 10).**

Efforts have been made to improve the quality of the above photos, unfortunately, these are the only photos we have.

**Reviewer #1**

**Line 52, there is double parenthesis**

The change was considered.

**Some of Latin names are in italics and some not. It should be unified.**

The change was considered.

**Line 206, double dot**

The change was considered.

**Line 227, the sentence should be changed. There is: “In results reported… it was reported.**

The change was considered.
